# Navigating the complexity of a collaborative, system-wide public health programme: learning from a longitudinal qualitative evaluation of the ActEarly City Collaboratory

**DOI:** 10.1186/s12961-024-01227-2

**Published:** 2024-10-02

**Authors:** Laura Nixon, Laura Sheard, Jessica Sheringham, Amy Creaser, Halima Iqbal, Patience Gansallo, Liina Mansukoski, Maria Bryant, Bridget Lockyer

**Affiliations:** 1 Wolfson Institute of Population Health, Queen Mary University of London, London, United Kingdom; 2 Department of Health Sciences, University of York, York, United Kingdom; 3 Institute of Epidemiology and Health Care, University College London, London, United Kingdom; 4 Bradford Institute for Health Research, Bradford Teaching Hospital NHS Foundation Trust, Bradford, United Kingdom; 5 School of Psychology, University of Leeds, Leeds, United Kingdom; 6 Faculty of Health Studies, University of Bradford, Bradford, United Kingdom; 7 Hull York Medical School, University of York, York, United Kingdom

**Keywords:** Public health, Interdisciplinary, Evaluation, Systems, Qualitative, Child health, Inequalities, Collaboration, Consortium, Programme management

## Abstract

**Background:**

Addressing the upstream social determinants of health (e.g. built environment, education) can reduce the burden of non-communicable diseases. To do so effectively often requires system-wide collaboration. However, collaborating across multiple sectors, organizations and disciplines within a complex system can be challenging. ActEarly was a public health research consortium that aimed to improve child health by building an interdisciplinary, cross-city partnership to develop and/or evaluate upstream interventions, increase research capacity and improve collaboration between researchers, local authorities and communities. This paper explores ActEarly’s experiences of navigating complexity to identify mechanisms that supported its implementation and proposes recommendations for future intersectoral and interdisciplinary population health research collaborations.

**Methods:**

We conducted a longitudinal qualitative study of ActEarly, integrating findings from inductive documentary analysis of internal documents (mainly meetings minutes and reports) (*n* = 114) and interviews (*n* = 70) with 45 consortium members at three different timepoints (2018, 2021, 2023). Participants worked across different organizations, cities, roles and levels of seniority in the consortium.

**Findings:**

Clarity, Unity, Flexibility and Feasibility were seen as the key mechanisms required to support ActEarly’s implementation. Clear aims, governance structures and communication were necessary to manage the uncertainty of the complex system. A unified approach, characterized by strong relationships, having a shared vision and communal access to resources supported effective collaboration. Flexibility was required to adjust to different ways of working, respond to wider system events and manage the consortium. Establishing feasible aims that responded to the limitations of the system, the available resources and research infrastructure was required for teams to deliver the work.

**Conclusions:**

Implementing multi-faceted programmes in a complex system can be challenging. We recommend that future whole-systems consortia seeking to improve population health build Clarity, Unity, Flexibility and Feasibility into their programmes, noting the complex interrelationships between these factors. Iterative reflections from all parties should support delivery amidst the uncertainty that comes with running a population health research collaboration, and strong leadership and governance should play a key role in ensuring that these are built into foundations the programme.

**Supplementary Information:**

The online version contains supplementary material available at 10.1186/s12961-024-01227-2.

## Background

It is recognized that addressing the social determinants of health (e.g. income, environment, education) plays a key role in reducing the burden of non-communicable diseases (NCDs) worldwide (1, 2). However, health inequalities are rooted in complex and long-standing societal problems that necessitate system-wide interventions and cross-sector collaborative approaches to address (3, 4). There are often no clear-cut solutions when it comes to tackling health inequalities, and attempting to target direct causal mechanisms in isolation can be seen as reductive and can have unforeseen consequences (5, 6). Taking a complex systems perspective can help to generate evidence that more fully considers the complex nature of real-world environments and subsequently increase impact (4, 7). Systems thinking requires researchers to consider the multiple ways in which an intervention might exert its effects and how those effects can be assessed. This means having to consider how to account for, and measure, a wide range of potential influences and outcomes outside of the researchers’ control (8). Attempting to predict the potential impacts of intervening in the system is further complicated when managing multiple simultaneous interventions; complexity theory underscores that no intervention operates in isolation and challenges the notion that one intervention can be understood as independent of previous and other concurrent interventions (6). As such, when attempting to influence population health, it is important to effectively collaborate across various parts of the system to ensure this complexity is considered and addressed in its entirety.

Recognizing the importance of systems approaches, collaborations have long been advocated to ensure policy decisions are evidence based and research is relevant (9–12). In recent years, interdisciplinary, cross-sector collaboration has been considered even more vital to meet the challenges of increasing health inequalities and to withstand major shocks to the public health system (13, 14). However, cross-sector collaboration can prove challenging, as collaborations themselves can be viewed as their own complex, dynamic, multi-level systems; this is further complicated by the need to work across multiple organizations with non-linear hierarchies (15, 16).

Academic institutions are traditionally organized into isolated research disciplines, and as such academics often face challenges working with others outside of their direct field of expertise (17, 18). Venturing outside of academia exacerbates these difficulties, and navigating working relationships between academics and policymakers is known to be particularly challenging. For example, research highlights different and/or competing interests; conflicting goals and timelines; language and terminology; working in isolated organizational, professional and disciplinary groups; unequal power dynamics; and mistrust between partners as barriers to successful collaboration between these groups (19–21). Research has explored these challenges, highlighted how existing contextual factors influence their success and suggested collaborative frameworks and strategies to address them (1, 3, 22–24). However, existing literature primarily focusses on individual aspects of collaborative challenges (e.g. cross-institutional but not interdisciplinary) and is not aimed at programmes that are attempting to manage many of these challenges simultaneously across multiple different intersecting systems. Understanding how to navigate this complexity has become more pertinent as the importance of collaboration in public health research starts to be more widely recognized, particularly in the UK context, where funders are increasingly investing collaborative, system-wide approach and boosting research capacity and capability within local government (25, 26).

The ActEarly City Collaboratory (2019–2025) was one such investment, a collaborative, system-wide population health research programme/consortium that worked across two areas of high deprivation in the United Kingdom (UK Prevention Research Partnership: MR/S037527/1). Taking a systems perspective, ActEarly aimed to build an interdisciplinary, cross-city partnership that worked together to build evidence to inform policy, develop upstream interventions and increase research capacity, with the ultimate goal of laying the foundations to improve child health and reducing long-term NCD risk factors in these high-risk areas (27). Recognizing the importance of system-wide collaboration and the challenges it created, ActEarly also aimed to break down some of these barriers and implement strategies which would create sustainable, long-lasting partnerships between academics, communities and local authorities (local government). The novel City Collaboratory approach was intended to take collaboration further by facilitating cross-site comparison and encouraging mutual support and knowledge sharing to promote the development of more “research-ready” cities. Made up of approximately 70 independently-funded projects and interventions across 5 thematic areas, 8 core organizations and 2 populations, ActEarly had to iteratively manage a high level of complexity in a dynamic system. Implementing a programme with so many different components and actors across many different parts of the system raises many challenges, even before taking into account the complexity and unpredictability of the wider system (16). Programme theory postulates that identifying the mechanisms that support programme implementation can help to navigate complexity and help to achieve the desired outcome (28).

This paper responds to the call for continued research and evaluation of multi-sector partnerships in complex systems, particularly those intentionally integrating policy, practice, community and research in collaborations for healthy public policy (29, 30). Using longitudinal qualitative data, it explores ActEarly’s experiences of navigating this complexity to identify the mechanisms that supported its implementation and propose recommendations for future inter-sectoral and inter-disciplinary population health research collaborations.

## Methods

### Study design

This paper describes a qualitative study which formed part of a larger, mixed-methods evaluation of the ActEarly collaboration (31). Here, we report on findings from the analysis of two sources of qualitative data: internal documents and interviews collected/conducted longitudinally over 5 years (2018–2023). Ethical approval was obtained from University College London Research Ethics Committee; wave 1 of the interviews were covered by UCL Ethics ID: 1037/003, while waves 2 and 3 of interviews and the documentary analysis were covered by UCL Ethics ID: 2037/004.

### Study setting/context

The settings of the ActEarly programme were the city of Bradford in the North of England and London Borough of Tower Hamlets (LBTH) in the South of England. Both areas have some of the highest health and wellbeing inequalities in England, a younger than average population and low disability-free life expectancies (32, 33). Bradford has more than 540,000 residents, a high Pakistani population (20%) and is the fifth largest metropolitan district in England (34). Child poverty is high, with 30% of children in absolute low-income families. LBTH has 310,300 residents, a third of whom are Bangladeshi, and its population is growing faster than any other area in the country (32). Additionally, 4 out of 10 households in LBTH live below the poverty line, and 32% of school children are persistently disadvantaged (32).

ActEarly comprised approximately 70 independently-funded projects and interventions, spanning across five main themes that targeted multiple areas in the system: Healthy Places; Healthy Learning; Food and Healthy Weight; Play and Physical Activity; and Healthy Livelihoods. These thematic areas were supported by two methodological, cross-cutting themes (co-production and evaluation), which provided consortium-wide project support and methodological capacity building. Within all these themes, academics partnered with a range of stakeholders across the two sites on a diverse set of projects spanning primary research, evaluation, intervention development/implementation, infrastructure development (e.g. data linkage) and community engagement events. For example, ActEarly worked with local authorities to improve whole-systems data linkage capabilities, worked with schools and authorities to increase uptake of free school meals, hosted regular knowledge exchange events in partnership with communities and voluntary organizations to set research agendas and alongside the NHS to improve access to routine data (e.g. primary care records) (31). Examples of ActEarly projects can be found in Appendix 1.

This complexity meant that the boundaries of the ActEarly system were fuzzy and changed over time. Reflecting the variety of ways ActEarly is described in both internal and external communications, we refer to ActEarly as a consortium, Collaboratory, and programme interchangeably in the text. More information on the programme can be found in Wright et al.’s (27) paper detailing the original ActEarly model (27).

### Sampling and data collection

#### Documentary analysis of internal ActEarly study documents

Our inclusion criteria for the collated documents was a universal sample of all formal internal documents produced during the 5-year project timeline between September 2018 and December 2023. We included 114 documents, relating to both site-specific meetings and joint meetings held between the two sites. Just under half the documents (*n* = 62) were formal minutes taken of remote and in-person executive group meetings attended by consortium members, either jointly across sites (attended by co-directors and theme leads) or independently (London: all staff; Bradford: senior staff). The rest were a mixture of formal reports to the funder (*n* = 6), minutes or reports from Scientific Advisory Group meetings (made up of public health specialists across charities, academia and local authorities) (*n* = 7), internal meeting minutes from staff workshop note (*n* = 18) and other relevant documents which did not fit into the above categories (e.g. internal updates, reports and logs) (*n* = 21) (Table 1).

**Table 1:** Breakdown of reviewed documents by type

Document type	Number
Joint ActEarly executive group meeting minutes	33
Bradford ActEarly executive group meeting minutes	12
LBTH ActEarly executive group meeting minutes	17
Annual reports and funder feedback	6
Board/ActEarly Scientific Advisory Group meeting minutes and reports	7
Other reports/documents (e.g. internal updates, reports and logs)	21
staff workshop notes notes	18
Total	114

#### In-depth interviews with ActEarly members

Interviews were conducted with ActEarly consortium members at three different time points: wave 1 interviews were conducted from September to October 2018, wave 2 from December 2021 to February 2022 and wave 3 from August to October 2023. Some of interviewees differed across the three cohorts due to new people joining the project and other people leaving over time. As such, 70 interviews with 45 members were conducted in total over the three waves [some participants were interviewed three times between 2018 and 2023 (*n* = 10), whilst others were only interviewed once (*n* = 29) or twice (*n* = 6)]. In wave 1, 13 participants were interviewed, wave 2 consisted of 20 participants and there were 37 participants in wave 3. Wherever possible we tried to maintain involvement of the same participants at each time point. We approached all directors (*n* = 2 at each timepoint, *n* = 3 total) and theme leads (*n* = 8) for interview; both directors were interviewed in each wave, 75% (*n* = 6) of theme leads participated in waves 1 and 2, and 87.5% of theme leads in wave 3 (*n* = 7). The rest of the participants were purposively sampled on the basis of their institutional affiliation, disciplinary background, study site and level of professional seniority. The eligibility of those without formal positions in the consortium was determined on the basis of the projects they were associated with and their level of engagement within the consortium. Due to the nebulous and dynamic nature of ActEarly’s boundaries, we do not provide total numbers for these groups. Table 2 presents a breakdown of participants by role and site (Table 2), and Table 3 a breakdown of participants by role and wave (Table 3). This paper refers to participants interchangeably between consortium members, researchers and staff. The word academics is used to describe those holding a research or teaching role at a higher-education institution [including co-directors, theme leads, and early career researchers (ECRs)]; researcher is used to describe anyone who works on research projects in any capacity; local authorities refers to those who work at the council/local government or affiliated services; and partners is used to describe individuals working at organizations external to academia or local authorities (e.g. charities, schools).

**Table 2:** Participants by role and site (all waves)

Role	LBTH	Bradford	Total participants
Co-directors	2*	1	3
Theme leads/co-leads	3	4	7
Co-investigators	5	4	9
Local authorities/partners	3	5	8
Project managers	1	3	4
Early career researchers	6	8	14
Total	20	25	45

^*^ LBTH co-director change after wave 1 added new participant

**Table 3:** Participants by role per wave

Role	Wave 1	Wave 2	Wave 3	Total individual participants
Co-directors	2	2	2	3
Theme leads/co-leads	6	6	7	7
Co-investigators	4	2	6	9
Local authorities/partners	0	2	7	8
Project managers	1	4	4	4
Early career researchers	0	4	11	14
Total	13	20	37	45

The topic guide for the interviews differed at each of the three timepoints and were developed for a wider mixed-methods evaluation of ActEarly’s overall progress and implementation (Appendix 2). In the 2018 and 2021 topic guides, questions primarily focussed on views on ActEarly’s contribution to interdisciplinary collaborations, its potential impact and sustainability and use of co-production and citizen science. The topic guide for the final wave was expanded to reflect ideas raised in previous waves and the newly-developed meta-evaluation questions (31), including further questions on linked data, impacts on decision-making and retrospective reflections on the programme’s successes and challenges (see Mansukoski et al. for more details) (31). The format of the interview questioning was flexible to allow participants to talk about what they considered to be important.

Participants were recruited predominantly via an email, with an attached information sheet and consent form. In wave 1, L.S. and J.S. recruited participants and they were interviewed by freelance researchers. In wave 2, B.L. and H.I. recruited and interviewed participants. In wave 3, L.N. and A.C. both recruited and interviewed participants. Interviews were conducted via a mixture of in person, video call or phone call and lasted approximately 45 min. All participants gave written informed consent, and all interviews were digitally recorded and professionally transcribed by an NHS-approved, secure transcription service.

### Analysis

The research question was developed inductively from reflecting on reports on previous analysis of the data. Brief internal reports of findings from reflexive thematic analysis (35) of interviews with consortium members had been produced at the end of each of the three interview waves (2018, 2022, 2023) to inform programme management. The first report was written by L.S. and J.S., the second by H.I. and B.L. and the third by L.N. The semi-inductive documentary analysis (*n* = 114) had been conducted and reported internally by L.N. (with oversight by L.S.) by following the stages proposed by Bowen (36) (skimming, reading and interpretation) with the broader meta-evaluation questions in mind (31).

In February 2024, the research team met for a day-long analysis workshop, where core themes depicted across all reports and sources were presented and additional avenues of qualitative investigation were discussed. The authors determined that exploring the challenges and enablers would serve as a starting point for further investigation. L.N. and B.L. subsequently returned to the data with this topic in mind, undertaking additional reflexive thematic coding across both datasets. This iterative process helped to finalize the research question and aim – to identify the mechanisms that support the implementation of a cross-disciplinary public health consortium. They then conducted further interpretive work to identify these mechanisms, sense checking with other authors where appropriate. L.S. had oversight of all analysis and reporting throughout the whole programme. We define the word mechanism as an organizational factor that affected consortium members’ ability to achieve their intended outcomes (i.e. delivering their work as planned), in either its absence or presence. The term is used in a conceptual sense, rather than intending to identify objective causal associations. Whilst our data were longitudinal in nature, we do not present it in a chronological manner in this paper. Rather, we use data sources from different timepoints to illustrate key points (with comparisons where appropriate). We decided to report on the integrated findings of datasets, as it was apparent to the research team that the themes contained within the documentary analysis report and the interview reports were similar, with each method providing supporting context that complemented the other dataset. The analysis conducted was manual without the use of a software package and wholly inductive, meaning we did not structure it on any existing theoretical frameworks.

## Findings

**Fig. 1:**
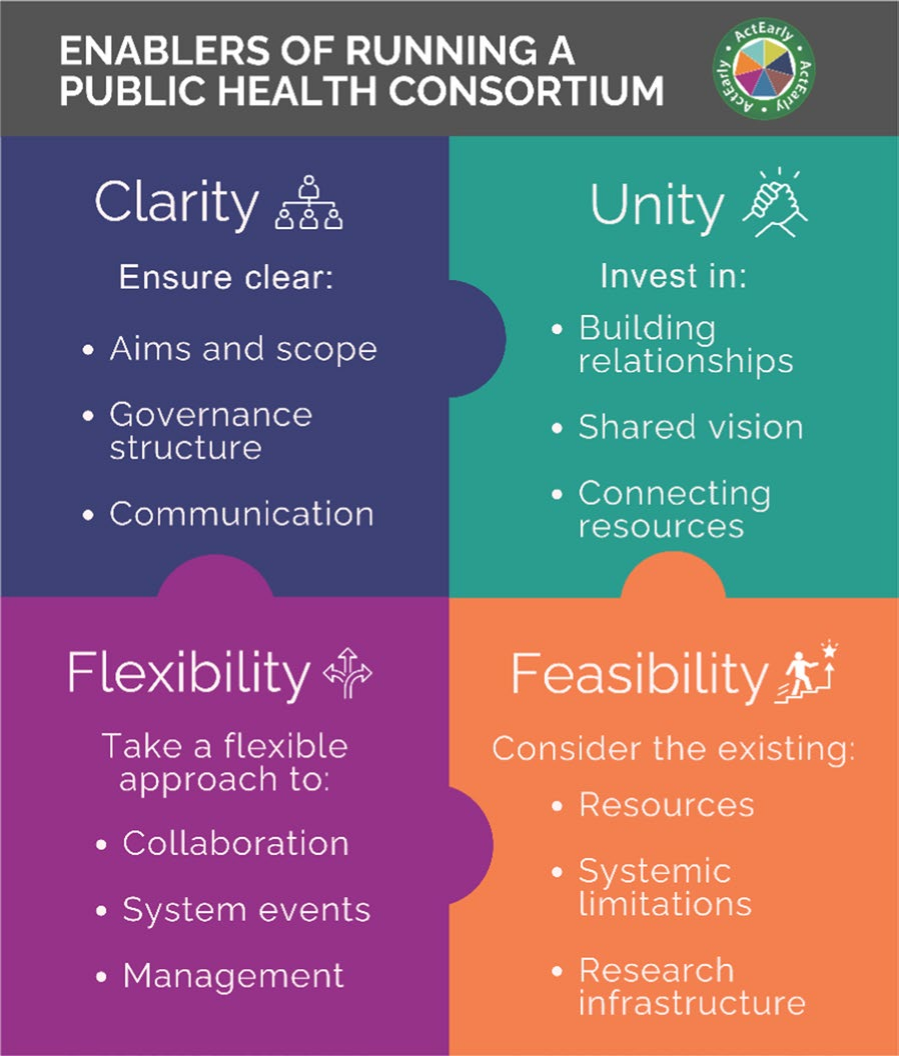
Enablers of running a public health consortium

Our analysis highlighted that implementing ActEarly raised many challenges and the extent that the programme was able to navigate these successfully was varied. By exploring accounts of how the programme handled these challenges and reflecting on participants’ recommendations, we identified clarity, unity, feasibility and flexibility as the key mechanisms likely to support the implementation of a public health consortium like ActEarly (Fig. 1).

### Clarity

Having clarity around the programme’s aims and scope, governance and communication was seen to enable consortium members to manage their work.

#### Aims and scope

Participants were in the latter stages of developing the grant proposal at the time of the wave 1 interviews, and thus were relatively confident in describing and discussing the programme’s aims to reduce NCDs and health inequalities through building collaborations, knowledge and research capacity. However, the clarity and structure of ActEarly’s aims became less clear as time went on and as more people joined the programme. When asked to define ActEarly and its aims in the second and third waves of interviews, many people were uncertain of its ultimate purpose, even those who had been confident in their previous answers in 2018:

“*I don’t think there is a complete clarity within the consortium about what ActEarly is about*” (P1, wave 2).

The promotional materials and interviews with ECRs tended to focus more on the programme’s aim of improving child health in areas of deprivation through building the evidence base, while those who had been involved since the start placed greater emphasis on aims that built capacity for future research.

Acknowledging these conflicting perspectives, consortium members believed that ActEarly “*could have defined the parameters much more*” (staff workshop notes notes, May 2023) and believed more tangible objectives would have helped to avoid confusion:

“*ActEarly is massive and it’s got very lofty aims but it would have been helpful to say you know, this is what we want to do and this is how we’re going to get there and then we sort of have steps that are actionable*” (P41, wave 3).

A logic model outlining the programme aims was available to the team from the start and was updated in 2022 for clarity and to reflect the refined goals. However, the perceived breadth of scope, the “blurred line” of what (and who) counted as part of ActEarly and inconsistent terminology in materials created some confusion. Participants did recognize, however, that a certain amount of ambiguity is unavoidable when taking a whole-systems approach:

“*from a sort of systems perspective, you don’t really have closed systems, so you can’t really say there’s a boundary around ActEarly and everything inside of that boundary is the project and everything outside is sort of straightforwardly not the project*” (P27, wave 3).

#### Governance

Consortium members wanted more clarity about governance structure, roles and an overarching plan throughout the lifetime of ActEarly. During meetings and interviews, staff sought clarity on the responsibilities and expectations of job descriptions, and some queried the purpose of the cross-cutting themes (evaluation and co-production), uncertainty about how to access resources and how links between sites operate:

“*the biggest issue that I find with ActEarly is understanding where the resources are and who ultimately is in charge of deciding how they should be best deployed*” (P7, wave 2).

This was acknowledged throughout the programme, and ActEarly’s Scientific Advisory Group requested that the team simplify the governance structure 2 years into the grant. Responding to some of these criticisms and to make these structures clearer, organograms depicting the programme’s organizational structure were developed and shared, and any confusion was addressed in meetings as specific challenges arose. Despite these remedies, participants still expressed uncertainty about governance structures and processes towards the end of the project. It was recognized that the scale and design of the programme made this challenging, but one participant emphasized that the complexity of the programme made strong governance even more essential and a “*necessary evil*” to ensuring project success:

“*a lot of it does come down to governance, right, and governance needs to include the forward planning, clarity about roles and responsibilities, where the decision-making points are, what the communication strategy is, a bit more oversight on how the money is being spent*” (P26, wave 3).

#### Communication

Clear and consistent communication was considered important to maintain relationships, avoid duplication of work and support project management. ActEarly having dedicated programme managers at each site and roles linking with the local authorities was considered to have been very useful in tackling communication challenges, especially in LBTH where academics were spread out across multiple institutions. Making sure there was regular communication happening at all levels was considered an important facilitator of project success:

“*it needs that constant dialogue and you know, it’s not enough to have it at the top or the bottom, it needs to be all the way through*” (P37, wave 3).

Participants found regular communication challenging when senior researchers had limited availability, which could hinder ECRs having access to project support, whether it be to get plans approved or guidance: “*something that might only be a 5-min conversation, it can actually take a week or so to arrange”* (P11, wave 2).

A lack of clarity around the boundaries of ActEarly led to confusion about who should be involved in mailing lists, meetings and updates, leaving some consortium members unaware of what was happening outside of their focus of work:

“*unless you’re kind of party to those governing spaces, you don’t necessarily know how other bits are working*” (P38, wave 3).

ActEarly tackled some of these issues by splitting some of the consortium meetings into smaller groups (e.g. by location or theme); the inclusion of less senior staff and key local government members supported communication locally and provided more of an opportunity to be involved in decision-making. However, it is important to note that this cross-site division may have also contributed to a disconnect within the wider consortium:

“*joining in the executive meetings at London level [...] really helped me connect a lot more, understand what was going on...However this has really created a complete divide from where I’m sitting between Bradford and London*” (P17, wave 3).

The introduction of e-bulletins in early 2022 was seen to mitigate some of the cross-site knowledge gap by providing a platform for consortium members to share their work and opportunities.

### Unity

Creating a sense of unity and cohesion between partners was considered a key facilitator of delivering the work, but was also felt by many to be the most challenging aspect of the programme to manage. It required investing in building and maintaining relationships, creating a shared vision and ensuring shared access to resources.

#### Relationships

Bringing together people from a range of disciplines, backgrounds and organizations was seen as a highlight of the programme, and building these collaborative relationships was considered an essential part of creating change:

“*you can get so far on your own sitting there observing ideas [...] but it’s only when you start sharing them with other people that you get the other pieces of the jigsaw and what evolves is something that none of us could have done our own”* (P1, wave 1)

Some people expressed that it was initially challenging to find the appropriate person to contact about a project. As well as being essential for supporting communication between existing partners, the development of roles linking the council and universities also made establishing new contacts in local authorities easier and in turn supported the development of new relationships:

*“in ActEarly, the most important thing has been to have a dedicated person in the local authority who facilitates contacts with other people in the local authority*” (P4, wave 2).

Having links to members of the public through partners such as voluntary and community sector organizations or schools also supported the building of researcher-community collaborations by being “*a bridge between the academics and local community and trying to find different ways to bring people together and have meaningful conversations*” (P30, wave 3). These partner organizations facilitated recruitment and held community events, which provided a platform for academics to connect with members of the public.

High staff turnover and the sprawling, cross-city nature of the programme made it challenging for many ECRs to feel part of the consortium. The addition of cross-site/cross-theme meetings, yearly week-long writing retreats and conference funding opportunities were considered to offer useful steps towards building these relationships: “*The writing retreat was also really helpful, especially for connecting with the guys outside of London […] it’s just a lot more like natural and organic to talk about projects*” (P39, wave 3).

Opportunities to connect with colleagues in person, such as workshops and conferences, also strengthened relationships across the consortium. At the same time, remote meetings allowed people to join meetings that they would never have been able to find time to attend, as well as keep in touch with colleagues across sites. Considerable investment in fostering a strong cross-site connection was needed, as the distance, competitive nature of academia and differences in working cultures made some feel like it was “*two kind of sites collaborating rather than a team collaborating across two sites*” (P16, wave 3).

Taking a systems approach was new to many, and participants acknowledged that it was challenging to find ways to collaborate effectively between so many different stakeholders who were used to working in a siloed way:

“*this is like wild west stuff you know, this is frontier stuff, multiple institutions, multiple disciplines, [...] working with a local authority that’s never really worked with academics before in an area that’s difficult enough to work in even if everybody was on the same page*” (P12, wave 2).

As such, it was recognized that creating strong collaborative partnerships required a significant amount of work and commitment to build and maintain:

“*you can’t overlook the investment of time required, the generosity of spirit required, the sharing of perspective, taking the time to build the relationships*” (P26, wave 3).

#### Shared vision and priorities

Commitment from all parties was seen as necessary to make relationships work, and cultivating a team mindset required investment:

“*you have to have good coordination. Keeping that synergy, keeping the sense that there’s a big prize here and we’re all in it*” (P3, wave 1).

Consortium members reported being driven by their shared passion for improving health inequalities, and having a “*unity of purpose*” (P42, wave 3) was seen as a motivating factor for, and facilitator of, developing these cohesive cross-site relationships. The importance of creating a shared vision and mentality between local authorities and academics was also acknowledged, with staff at a 2021 workshop noting that “*political mindset works on the idea we need to change something! Research is about finding out what the evidence tells us needs to change. This needs to come together and get a consensus*” (staff workshop notes notes, November 2021).

#### Connected resources

Our participants acknowledged that making it simpler to share data, resources and finances across partner organizations would have supported better cross-institutional collaboration. Restricted or delayed access to data was a major barrier to projects which disrupted project timelines and diverted researcher capacity:

“*a considerable amount of time and effort during these first two years of the project has gone into getting data sharing agreements in place*” (Healthy Places theme summary, 2021).

ActEarly worked to build this infrastructure and support data linkage through cross-site projects, supporting the development of “whole-systems data linkage accelerators” which participants hoped would make accessing data simpler in the future. Budgets were managed separately by independent finance departments at each institution, making accessing and keeping track of funds a time-consuming and complicated process. Having links to specific points of contact at each institution helped to manage this challenge.

Different institutions used different productivity and collaboration software (i.e. Google for Business versus Microsoft360) and had different data protection policies, which often made sharing research data or even internal documents a lengthy and complex process. One participant suggested that “*having good policies and procedures and support or even just directions for how you access the same information*” across institutions would support efficient cross-institutional working (P34, wave 3).

### Flexibility

To have an impact in public health, consortium members believed that “*researchers need to do research that is accessible, reactive and flexible for rapid implementation and responsive to change*” (staff workshop notes notes, November 2021). Flexibility was seen to facilitate the management of the external influences outside of the team’s control.

#### Collaboration

Participants felt that being flexible to adjusting to different working cultures and expectations across different disciplines, roles and institutions was vital:

“*you have to start to learn to adjust and to have ways of communicating and connecting people, that you recognize those cultural differences and different ways of working*” (P10, wave 2).

Overcoming language barriers and working to “*consider how to resolve conflict and conflicting agendas”* between different stakeholders (London AEG minutes, April 2023) were considered important to keep everyone on the same page. Local authorities tended to work on shorter timescales than academics, making expectations of what was achievable in the time frame “*difficult to manage*” (P2, wave 3), while academics were “*doing great things, but not necessarily what local authorities needed, or certainly not in the format that they needed it in*” (P1, wave 3). Acknowledging this difference and being willing to adapt to each other’s communication styles was seen to be important when addressing this disconnect.

Collaborating with the public also required a more flexible approach than other research, as the iterative and reflexive nature of working with communities can mean that “*you don’t know at the very start of the project exactly what you want to do*” (P10, wave 3).

#### Wider system events

Events occurring outside of the direct ActEarly system affected programme implementation both directly [e.g. coronavirus disease 2019 (COVID-19)] and indirectly (e.g. shift in political priorities). ActEarly was willing to adapt its processes to meet the needs of various wider system events that affected the running of the programme:

“*what we’ve learnt as we go along is how to make it work in practice, so where you have to be flexible and where you have to sort of fit with local politics or local resources*” (P10, wave 2).

The COVID-19 pandemic and ensuing lockdowns were a major disruption that required consortium members to adapt their research priorities and approaches. One participant reflected that adjusting to these changes was about “*having to compromise, having to be quite pragmatic*” (P36, wave 3) about what could be achieved. Many quickly adapted to the disruptions by redirecting their research efforts towards pandemic-related research. This agility allowed them to provide timely evidence to support local initiatives, and in some cases acted as a springboard for the development of new research. For example, a study on the provision of food aid in Bradford during the pandemic provided rationale for a wider review of community food assets across both sites.

Consortium members had to consider the political acceptability of their work and dissemination to maximize the chance of it making an impact. Changes in political leadership meant some projects were disrupted or cancelled when they did not fit with the new political agenda. This was particularly challenging for work around street safety, as low traffic neighbourhoods became a highly divisive issue in LBTH. It was recognized that “*politics can’t be planned for in advance*” (staff workshop notes notes, May 2023), but meetings regularly addressed how researchers “*mustn’t underestimate the political interface of their work*” (London AEG minutes, April 2023) and should iteratively reflect on “*where there might be linkages with the activities in the political cycle*” (staff workshop notes notes, March 2022).

Working within a wider system did not just mean considering the impact external events had on the programme, but being prepared to adapt plans to accommodate the unpredictable impact the programme’s actions on the system: “*I think it’s a big challenge because when we pull a lever here we don’t always know like how it reverberates throughout the whole system*” (P13, wave 2).

#### Programme management

The large number of stakeholders in the consortium meant the programme frequently had to manage internal changes. Staff turnover and maternity leave commonly disrupted capacity and influenced the strength of interpersonal relationships over time. Leadership took a flexible approach to managing these challenges:

“*there’s so many people involved it’s its own complex adaptive system. So there’s a bit about not trying to control it or trying to fix it too much, but let it happen*” (P5, wave 3).

Participants felt that this more decentralized management structure had given them the freedom to respond to wider system changes:

“[ActEarly’s] philosophy is a lot more actually people should be self-organizing […].

That’s really exciting and it enables you to respond more agilely to Council agendas” (P32, wave 3).

Though not involved in the day-to-day of individual projects, leadership was still regularly updated and led the management of unexpected circumstances and challenges. The programme was open to hearing and adapting to staff concerns; interviews with consortium members halfway through the programme informed changes which aimed to support building relationships, improving communication and providing training opportunities.

Despite the challenges of managing ActEarly’s ‘fuzzy boundaries’, some also saw this versatility as one of the programme’s strengths, as it allowed for synergistic relationships with different programmes and opened up wider opportunities: “*I think there is some benefit in it being loosely defined sometimes because then you’re not constrained*” (P16, wave 3).

### Feasibility

The breadth and scale of ActEarly’s aims were considered too ambitious by many, and a result of having to “*promise the world to get the funding*” (P2, wave 3). Participants emphasized that it would be important to consider the feasibility of achieving a programme’s aims within systemic limitations, available resources and existing infrastructure when developing similar programmes in the future.

#### Resources

Having sufficient funds and staff capacity were seen to contribute to the feasibility of achieving the programme’s aims. However, many participants believed that ActEarly had been undercosted for what it set out to achieve:

“*a few million quid over five years, split between two areas in billion pound economies, you know, you’ve got to be realistic about what to expect in terms of outcomes*” (P5, wave 3).

As a grant based on facilitating collaboration, it relied on members independently gaining funding for their projects. This meant that the cost of interventions was not covered, which disrupted the initial plan to focus on evaluating them.

Sufficient researcher capacity was highlighted as being vital to being able to deliver. The annual report in 2021 recognized that ActEarly’s interdisciplinary, whole-systems approach meant that it was important to have expertise across the “*range of disciplines needed to cover the broad areas important to wider determinant NCD research*”, but that doing so it “*significantly spreads the resource thinly leaving many with very limited funded time*” (annual report, 2021). Some themes only had one ECR working across both sites, often part time. Expectations around what ECRs could achieve with this limited capacity were not considered realistic:

“*a singular research fellow on two days a week is not a realistic amount of work capacity for what the project aims to do*” (P34, wave 3).

Participants commonly suggested that the work would have been more feasible if the programme had funded fewer co-investigators but with a higher time commitment, as well as more “on-the-ground” research staff:

“*We funded the grant in a typical way, which is lots of co-apps at 5% and what we should have done is [...] fund more research fellows, more research management capacity at the beginning and have a much more agile way of funding researcher input”* (P32, wave 3).

#### Systemic limitations

Working within multiple overlapping systems (e.g. academic, governmental) meant delivering objectives within the limitations of regulations, culture, timelines and expectations. Participants recommended considering these wider constraints when determining whether an activity is feasible. Co-production was considered a priority for ActEarly; however, funder timelines, administrative barriers and a lack of researcher experience limited the extent that it could be incorporated. Including communities meaningfully in research required time and flexibility, and funders did not like unclear research questions. It was also believed that the current system made it challenging to receive ethical approvals and pay external partners/community groups:

“*you need to design [the system] in a way which is going to make it easy for communities to get involved. Currently, it’s not.*” (P12, wave 3).

Achieving true interdisciplinarity was felt to be hampered by systemic and academic boundaries. For example, participants found that it was difficult to be awarded grants outside of their primary academic discipline:

“*if the funder is a funder with a very strong health focus then I do find that is a lot better that the lead is in fact a health researcher or academic*” (P7, wave 2).

A period of 5 years was not considered to be long enough to achieve the building of sustainable infrastructure, networks, and a measurable impact on population health. It was noted that acknowledging that this was not feasible was important when measuring the programme’s success, and that smaller impacts should not be seen as failures, but as progress towards the wider high-level aims of public health research:

“*This is societal stuff, it’s big stuff you know so you have to move boulders to do that and we’re moving stones at the moment you know so, but the stones add up to boulders eventually*” (P12, wave 2).

#### Research infrastructure

The availability of research infrastructure affected the feasibility of the work. Bradford started the project with a stronger research infrastructure across data linkage, social networks, community links and research culture. LBTH faced more challenges in executing their plans as a result, and consortium members believed it was useful to consider that the difference in infrastructure could mean that different sites could need different timelines, funding and expectations. ActEarly was designed to build on the existing infrastructure in Bradford, and thus was prepared to manage this difference, though it still took more time and investment than many had originally thought. Workshops, projects to identify challenges, and regular engagement at all stages of the research process was believed to help to overcome these limitations. Several suggested that accounting for additional time to lay the foundations for future collaboration would increase the feasibility of similar work:

“*I think in some ways you almost need a two-year lead time to develop relationships, to ascertain and get access to data and to data confidentiality agreement*” (P8, wave 3).

## Discussion

This paper draws on analysis of evidence from 70 interviews and 114 documents collected over 5 years to provide insights into the complexity of running of a whole-systems programme that was attempting to simultaneously strengthen interdisciplinary, cross-city collaborations, build capacity and infrastructure, develop new knowledge and effect policy change within and across a web of networks with differing levels of capacity (27). Our analysis highlighted four key mechanisms that were seen to support the programme’s implementation: clarity, unity, flexibility and feasibility (CUFF). When accounted for effectively, CUFF enabled the programme to achieve its aims. However, ActEarly did not always successfully apply these CUFF principles, and many of our findings were based on what was learnt when navigating these setbacks.

Consortium members felt that clear communication and well-defined aims, plans and governance structures facilitated their work, but that the programme’s complexity made this particularly challenging. Unity was considered vital in developing effective and innovative solutions to public health challenges; building strong relationships, having a shared vision and joint access to resources facilitated effective collaboration. Establishing “link” roles between networks and having dedicated programme managers were seen to help to build this Clarity and Unity. Ensuring that project teams have access to sufficient resources and research infrastructure to feasibly achieve their aims was widely considered a priority, as many felt that being under-resourced had been a major barrier to their work. Participants believed that it was important to be realistic and fully consider the limitations of the system when assessing what resources are needed. Flexibility was seen as an important strategy to manage the external influences that threatened the clarity and feasibility of the programme’s work, to adapt to the unpredictable system events and to facilitate stronger collaboration.

A year into running similar capacity-building public health collaborations, local authorities in the United Kingdom are also reporting similar challenges to ActEarly when building capacity and relationships, recommending that similar endeavours should not underestimate the importance of strong leadership and sufficient capacity and resources (37). The challenges ActEarly faced also mirrored that of other complex programmes and interdisciplinary collaborations in the literature (18–21, 38). This paper identified ways to mitigate these challenges, and our recommendations align with previous studies which have identified factors such as synergies in rapport (trust, comfort and openness); commitment to, and faith in, the project; equalities in capacity and resources; using common language; quality of connections; interorganizational communication; and existing infrastructure as facilitators of success (10, 23, 39–41).

Our findings particularly resonate with the work of de Montigny et al., whose framework for cross-sector collaboration highlights the importance of collective planning, motivation, capacity and adaptability when working towards improving population health (3). An equitable approach to decision-making is also commonly reported to improve collaborative practice (3, 24, 40, 42, 43). Though this was not an explicit theme in our analysis, participants addressed this indirectly through their emphasis on communication, accommodating for working culture, building relationships and valuing decentralized management.

Systems and project management literature supports our argument for the importance of being able to react flexibly to emergent events (44), and many other complex “mega-projects” in other industries are moving away from “plan and control” management approaches towards “organizational improvisation” to mitigate for external changes that might influence the effectiveness of the work (44–46). At the same, more hands-off approaches have been associated with reduced productivity, motivation and efficiency (47, 48), and instigating frequent change in a project can make roles and aims even less clear (38). Although we did not find evidence of low productivity or motivation, our participants did raise concerns about the lack of guidance and clarity that came as a consequence of the programme’s flexible approach. This highlights the importance of promoting flexibility and worker autonomy while maintaining clear communication and governance.

Programme outcomes may be determined by context and planning needs to take system limitations into account (16, 49); our analysis supported the notion that management also needs to iteratively consider wider contextual factors (such as available infrastructure, system limitations and system events) to support programme implementation.

### Strengths and limitations

The quantity and depth of our longitudinal data provided the opportunity to get a thorough insight into people’s experience of being a part of the consortium and the decisions that were made across the life of the project. This breadth substantiated the generalizability of our findings across multiple situations (e.g. different stages of the programme, pre- and post-COVID-19) and benefitted by not simply being reported by participants in hindsight.

The data were collected, analysed and reported by funded members of the consortium, and the wave 3 interviews took place while participants were still employed by the programme. This could have meant that interviewees were uncomfortable sharing any negative views and it was more challenging to minimize author bias. However, we found that participants were willing to critically reflect on the failures as well as the successes; many of our findings came from what they believed would have strengthened the programme rather than solely from participants “selling” their achievements. The size of our sample and the number of authors involved in handling the data also helped to minimize bias, as any claims had to be substantiated by considerable evidence and supported by author consensus. Voices from the community were not included in the interviews due to our focus on the internal organizational functioning of the programme. However, participants did include members of voluntary and community organizations (*n* = 2) and experts in community engagement research (*n* = 5), and further evaluative work is being undertaken focussing on this aspect of ActEarly’s implementation. Although our data were longitudinal, we did not emphasize the temporality of the data, as we found that challenges were relatively consistent throughout the journey. Most other literature on collaborative working has focussed on individual facets of running a system-wide, population health consortium and/or are reviews or commentary papers rather than longitudinal qualitative analysis. We add to this body of research by highlighting supportive organizational mechanisms that apply across multiple contexts and provide researchers with both a high-level set of considerations that could be applied to multiple contexts, be it physically (e.g. location), culturally (e.g. organization) or temporally (e.g. project stage, wider events), while also breaking our findings down into practical recommendations more specific to programmes such as ActEarly (Fig. 1).

### Implications and recommendations

This paper explored the mechanisms that were key to ActEarly’s journey, notable either in their presence or absence, and highlighted the importance of considering CUFF when attempting to implement such a complex programme in such a complex system. To navigate this complexity, we recommend iteratively reflecting on the clarity of aims, structures and communication; investing in building relationships, developing a shared vision, and connecting resources; taking a flexible approach to collaboration, system events and management; and realistically assessing the feasibility of goals within the limitations of the existing resources, system limitations and infrastructure (Fig. 1). However, like the system itself, we found that the relationships between the CUFF mechanisms were complex, and any intervention to support one aspect of the programme is likely to have a knock-on effect (6). For example, increasing the number of people involved in the programme and amending plans affected clarity of communication and certainty, while taking on new work during the pandemic spread staff capacity and resources more thinly. When making reactive decisions, it is therefore important to consider how any changes may disrupt previous efforts to build CUFF into the programme. Though senior leadership may be best placed to ensure these factors are considered, we believe that colleagues across all organizations and levels of seniority would benefit from considering CUFF, particularly when it comes to supporting regular communication and engagement with the wider consortium. Other actors in the system also play a role in supporting the success of such projects. We encourage funders to consider that, for their grants to have the potential to be transformative in the future, the goals of the projects they fund must be achievable during the grant period and that supporting the flexible use of funds may help to ensure the delivery of the work.

Interdisciplinary, systems-based collaborations are vital in improving population health and reducing health disparities (3, 4), and previous research has recommended that first-hand evidence from consortia such as ActEarly should play an important role in the design of future intersectoral health collaborations (30). Though complex programmes and interventions are difficult to evaluate (7, 49), it is important for researchers to continue to record and evaluate their experiences to build up the shared knowledge necessary to maximize future impact. This paper reports findings that are a valuable part of this capacity building, and we hope that sharing them will support others to build on these foundations.

## Conclusions

Ensuring clarity, unity, feasibility and flexibility can help researchers across different organizations, disciplines and levels of seniority navigate the uncertainty that comes with delivering a complex collaborative public health programme. Strong leadership and governance play a key role in ensuring that these are built into foundations the programme. We hope that these insights will support future consortia and whole-systems programmes in their endeavour to improve population health.

## Supplementary Information

Additional file 1.

Additional file 2.

## Data Availability

The datasets used and/or analysed during the current study are available from the corresponding author on reasonable request.

## Abbreviations

NCD: Non-communicable disease
LBTH: London borough of Tower Hamlets
ECR: Early career researcher
P: Participant
CUFF: Clarity, unity, flexibility and feasibility
