# Supplementary material for: Navigating the complexity of a collaborative, system-wide public health programme: learning from a longitudinal qualitative evaluation of the ActEarly City Collaboratory
Source: Health Res Policy Syst. 2024 Oct 2;22:138. doi: 10.1186/s12961-024-01227-2 (PMC11446050; doi:10.1186/s12961-024-01227-2)
Supplement: Supplementary file 1 — Additional file 1. [file 12961_2024_1227_MOESM1_ESM.docx]

| **Appendix 1: Example ActEarly projects** | | | | |
| --- | --- | --- | --- | --- |
| **Project title** | **Population** | **Aim** | **Study Type** | **Study site** |
| Green Space development and evaluation | Adolescent girls | 1. To understand how adolescent girls use local greenspaces and what influences their behaviours. 2. To evaluate the feasibility and acceptability of co-designed greenspace development with adolescent girls 3. To evaluate the impact of co-designed greenspace development on greenspace usage and quality | Mixed-methods (focus groups, survey) | Bradford |
| ClassACT (Covid Air Disinfectant System) | Primary schools | i) To evaluate the feasibility and practical implementation of air cleaning technologies in a primary school context; and ii) to assess the effect of air cleaning technologies applied as a widespread intervention on transmission of the SARS-CoV-2 virus and other infections (including infection-mediated respiratory illnesses) in school settings. | Quasi Randomised Control Trial | Bradford |
| Whole system data linkage accelerators: a North-South partnership to unlock public health data | N/A | i) To describe the experiences and barriers of data system development based on our experience with Connected Bradford and the Tower Hamlets Whole Systems Project. ii) To explore the views of data users, stakeholders and community members through qualitative focus groups and interviews. iii) To map data flows required to develop useable data pathways including timing and coverage of data capture, nature of electronic data recording, sharing of data across systems, capture of repeated measures. iv) To develop a business case for sustainable linkage to inform decision making and compile an Implementation Guide for local authorities. | Mixed methods (case study, interviews, linking data) | Bradford and Tower Hamlets |
| Fast-food Exposure and Childhood Obesity in Tower Hamlets and Bradford | Children aged 5-12 years | To explore the relationship between exposure to fast food outlets and home, school and journeys between these and childhood obesity in children aged 5 to 12 | Longitudinal secondary data analysis | Bradford and Tower Hamlets |
| Healthy School Streets in TH and Bradford | School-aged children | To assess the impact of 'school streets' on i) active travel to school, ii) satisfaction with the journey to school and the street outside the school, iii) self-reported health and wellbeing. | Natural experiment | Bradford and Tower Hamlets |
| Play in Urban Spaces for Health | Pre-school and primary-aged children in urban environments | i) To investigate how policy and environment can be influenced in regenerating urban areas to ‘design-in play’; ii) explore the potential for an PUSH programme for 3-7 year olds to be delivered by schools; and iii) identity suitable tools for measuring physical activity, play, and wellbeing outcomes of pre-school and primary aged children including those with special educational needs within the school setting and urban environment. | Qualitative (interviews, focus groups, workshops) | Bradford and Tower Hamlets |
| Inequalities of access to Early Years Care and Education in Tower Hamlets and Bradford: mapping, focus groups and interviews | Parents and pre-school aged children | i) To map and quantify access to early childcare services in Tower Hamlets and Bradford, aiming to reveal underlying inequalities and guide the strategic planning of future service distribution in TH. | Mixed-methods (mapping, focus groups, interviews) | Bradford and Tower Hamlets |
| Synergies between housing and local environments for enhanced child wellbeing in Tower Hamlets and Bradford | Parents of school-aged children | i) To explore how parents feel about the quality of their home and local outdoor environments and community assets ii) To assess if there is complementarity between housing and neighbourhood-level aspects which could support (or hinder) their children’s health and wellbeing | Qualitative (Interviews) | Bradford and Tower Hamlets |
| Nothing about us without us: Developing a co-production strategy for communities, researchers and stakeholders to identify ways of improving health and reducing inequalities | Adults in Bradford and Tower Hamlets | To co-develop a co-production strategy to provide guidance on how communities, researchers and stakeholders can work together to identify ways of improving health and reducing inequalities | Qualitiative (workshops, interviews, focus groups) | Bradford and Tower Hamlets |
| The effectiveness evaluation of the Join Us: Move Play (JU:MP) programme | Children aged 5-11 years | To assess the effectiveness of JU:MP, a whole-system physical activity intervention for children aged 5-11 years old, upon increasing moderate to vigorous physical activity after 24 months and 36 months of intervention . | Quasi-experimental (non-equivalent groups design study) | Bradford, Leeds, Wakefield, Calderdale, Shefiedl, Kirklees |
| Inequalities of Access to Early Childhood Provision: the case of Early Learning at 2 in Tower Hamlets | Providers of early education and care, community organisations, and parents of young children | To explore why uptake of Early Childhood Care and Education and the 'Early learning at two' offer was reduced post-pandemic | Qualitative (interviews) | Tower Hamlets |
| Free School Meals Quantitative Evaluation in Primary Schools | Year 6 pupils | To assess the impact of the discretionary UFSM schemes on i) education attainment and school absence; ii) weight status; iii) healthcare utilisation | Natural experiment | Tower Hamlets, Newham, Southwark, Islington |
