# Supplementary material for: Navigating the complexity of a collaborative, system-wide public health programme: learning from a longitudinal qualitative evaluation of the ActEarly City Collaboratory
Source: Health Res Policy Syst. 2024 Oct 2;22:138. doi: 10.1186/s12961-024-01227-2 (PMC11446050; doi:10.1186/s12961-024-01227-2)
Supplement: Supplementary file 2 — Additional file 2. [file 12961_2024_1227_MOESM2_ESM.docx]

**Topic guide 1 – Waves 1 and 2**

Researcher to introduce themselves and the rationale for the study. Check they have read the information sheet and emailed the consent form back. Include an assertion of anonymity and confidentiality.

Researcher to ask brief details about participants role, disciplinary background and academic or clinical responsibilities in order to frame the interview.

**Questions directed at participants who have not been interviewed before**:

- Can you tell me how you came to be involved in ActEarly? Who introduced you/contacted you?
- Could you describe the ActEarly theme you are leading? In what ways does this theme relate to other the themes (or not)?

**Questions for all participants:**

- Tell me about the collaborations that you have formed with other people as a result of ActEarly. How were these formed and how are they maintained?
- What (or who) did you find most effective for forming the consortium links? What challenges/constraints do you think the consortium may face/has already faced in this respect?
- What are your thoughts on the sustainability of ActEarly? (in the medium to long term)
- How far do you think the ActEarly is working in a transdisciplinary way? Are there areas for improvement?
- How would you describe the main aims of ActEarly? How well are the consortium working towards achieving these aims?
- How embedded are co-production and citizen science within your theme?
- What do you think the impact of ActEarly will be on population level changes? Why do you think this?
- How far do you feel guided by a whole systems-approach within your theme?
- Any other thoughts on the topics above? Anything we have not discussed but you would like to? Any subject you would like to return to?

**WAVE 3**

**Topic guide 2 – Wave 3 (Staff)**

Introductory questions

Researcher to ask brief details about participants role, disciplinary background and academic or clinical responsibilities in order to frame the interview.

- Can you tell me a bit about your role?
- When did you become involved in the programme?
- How do you feel about your role and how it fits into ActEarly as a whole?
- *Question for those not interviewed prior (as part of RoR):* Can you tell me how you came to be involved in ActEarly? Who introduced you/contacted you?

Knowledge and understanding of ActEarly as a whole

- How would you describe ActEarly? What would you say are the main aims of ActEarly?
- How is ActEarly working towards achieving these aims?
- What do you think is working well?
- What do you think hasn’t worked as well?

Implementation of ActEarly themes

- Could you describe the ActEarly theme you are leading or working as part of?
- In what ways does this theme relate to other ActEarly themes?
- In what ways does your theme work differently to other ActEarly themes?
- What studies or interventions have been implemented in your ActEarly theme?
- Was this implemented as initially intended or expected?
- Could you tell me about any contextual factors that may have influenced this?
- Were studies and interventions in your ActEarly theme implemented differently to how they were intended or expected, in what ways and why?
- Could you tell me about any contextual factors that may have influenced this?

Collaboration and capacity

- How much have you worked with the other site?
- Have you formed any collaborations with other people as a result of ActEarly? Tell me about how ActEarly has influenced your capacity to work with others outside your organisation.
- How were these formed and how are they maintained?
- What (or who) did you find most effective for forming the consortium links? What challenges/constraints do you think the consortium may face/has faced in this respect?
- How far do you think ActEarly is working in a transdisciplinary way? Are there areas for improvement?

Co-production and citizen science

- Are you aware of the ActEarly co-production and citizen science strategy? How embedded are co-production and citizen science within your theme?
- *If they are embedded:* how did embedding co-production and citizen science within your theme change the work carried out in your theme?
- *If they are not embedded:*  what have been the challenges of doing this? How could embedding co-production and citizen science within your theme change the work carried out in your theme?

Intervention evaluations

- Are you aware of any ActEarly intervention evaluations within your theme?
- Do you think the evidence obtained from these has been acted on, and how? Has there been investment in interventions, adaptation of existing interventions or the discontinuation of interventions?

Linked datasets

- Are you aware of the use of linked datasets as part of your role?
- If yes, do you think ActEarly has contributed towards the linkage between local datasets? If so, to what extent, and how?

Decision making

- Are you aware of any decisions that ActEarly research has influenced at a local or national level?
- If so, what decisions have been made because of ActEarly, to what extent, and how?

Outcomes of ActEarly

- Do you think ActEarly has created population level changes?
- What/Why do you think this?
- What are the long-term goals of the current work you are conducting?
- What are your thoughts on the sustainability of ActEarly? (in the medium to long term). Why?
- What challenges have affected your work?
- What do you think has worked well within ActEarly? What could have been improved?
- How do you think the work should be taken forward?

Concluding remarks

- What have you personally taken from the project?
- Any other thoughts on the topics above?
- Anything we have not discussed but you would like to?
- Any subject you would like to return to?

**Topic guide 3 – Wave 3 (Partners)**

Introductory questions

Researcher to ask brief details about participants role, disciplinary background and academic or clinical responsibilities in order to frame the interview.

- Can you tell me a bit about your role?
- When did you become involved in the programme?
- How do you feel about your role and how it fits into ActEarly as a whole?
- *Question for those not interviewed in prior waves:* Can you tell me how you came to be involved in ActEarly? Who introduced you/contacted you?

Knowledge and understanding of ActEarly as a whole

- How would you describe ActEarly? What would you say are the main aims of ActEarly?
- How is ActEarly working towards achieving these aims?
- What do you think is working well?
- What do you think hasn’t worked as well?

Partner involvement

- What involvement do you have with ActEarly as part of [partner organisation]? How does your role relate to ActEarly?
- If their role involves implementing ActEarly components, ask: What has been implemented as part of ActEarly in [partner organisation]? Were they implemented as initially intended or expected? Could you tell me about any contextual factors that may have influenced this.
- What things were implemented differently to intended or expected, and why? Tell me about any contextual factors that may have influenced this.

Collaboration and capacity

- Tell me about your experience working with others outside your organisation as a result of ActEarly.
- Have you worked with people you would not have typically worked with as a result of ActEarly (such as other partners or organisations, researchers, practitioners)?
- Have you formed any collaborations with other people as a result of ActEarly? Tell me about how ActEarly has influenced your capacity to work with others outside your organisation.
- How were these formed and how are they maintained?
- What (or who) did you find most effective for forming the consortium links?
- What challenges/constraints do you think the consortium may face/has faced in this respect?
- How far do you think ActEarly is working in a transdisciplinary way? Are there areas for improvement?
- Are there any areas that ActEarly could improve when working with [*organisation*]?

Co-production and citizen science

- Are you aware of the ActEarly co-production and citizen science strategy? How embedded are co-production and citizen science within your theme?
- How well do you feel like ActEarly engages stakeholders, members of the public or target users in the work they are doing?

Intervention evaluations

- Are you aware of any ActEarly intervention evaluations?
- Do you think the evidence obtained from these has been acted on, and how? Has there been investment in interventions, adaptation of existing interventions or the discontinuation of interventions?

Linked datasets

- Are you aware of the use of linked datasets as part of your role?
- If yes, do you think ActEarly has contributed towards the linkage between local datasets? If so, to what extent, and how?

Decision making

- Has the evidence produced by ActEarly changed anything about the decision made in your [organisation]? To what extent, and how? If not, why do you think this?
- Are you aware of any decisions that ActEarly research has influenced at a local or national level?
- If so, what decisions have been made because of ActEarly, to what extent, and how?

Outcomes of ActEarly

- Do you think ActEarly has created population level changes? What/Why?
- What are the long-term goals of the current work you are conducting?
- What are your thoughts on the sustainability of ActEarly? (in the medium to long term). Why?
- What challenges have affected your work?
- What do you think has worked well within ActEarly? What could have been improved?
- How do you think the work should be taken forward?

Concluding remarks

- What have you personally taken from the project?
- Any other thoughts on the topics above?
- Anything we have not discussed but you would like to?
- Any subject you would like to return to?
